# Prevalence of fungal DNAemia mediated by putatively non-pathogenic fungi in immunocompromised patients with febrile neutropenia: a prospective cohort study

**DOI:** 10.1186/s13045-024-01583-0

**Published:** 2024-08-07

**Authors:** Chantal Lucini, Klára Obrová, Isabella Krickl, Filomena Nogueira, Iva Kocmanová, Susanne Herndlhofer, Karoline V. Gleixner, Wolfgang R. Sperr, Tijana Frank, Nuno Andrade, Christina Peters, Gernot Engstler, Michael Dworzak, Andishe Attarbaschi, Martine van Grotel, Marry M. van den Heuvel-Eibrink, Ivan S Moiseev, Yuliya Rogacheva, Ludmilla Zubarovskaya, Natalia Zubarovskaya, Herbert Pichler, Anita Lawitschka, Elisabeth Koller, Felix Keil, Jiří Mayer, Barbora Weinbergerová, Peter Valent, Thomas Lion

**Affiliations:** 1 St. Anna Children’s Cancer Research Institute (CCRI), Zimmermannplatz 10, Vienna, A-1090 Austria; 2 Department of Clinical Microbiology and Immunology, University Hospital Brno, Brno, Czech Republic; 3 Department of Internal Medicine I, Division of Hematology and Hemostaseology, Medical University of Vienna, Vienna, Austria; 4 Ludwig Boltzmann Institute for Hematology and Oncology, Medical University of Vienna, Vienna, Austria; 5 Department of Pediatrics, St. Anna Children’s Hospital, Medical University of Vienna, Vienna, Austria; 6 Princess Máxima Centre for Paediatric Oncology, Utrecht, the Netherlands; 7 Division of Childhealth, Wilhelmina Childrens Hospital, University of Utrecht, Utrecht, the Netherlands; 8 RM Gorbacheva Children Research Institute, Pavlov University, Saint Petersburg, Russian Federation; 9 3rd Medical Dept, Hanusch Hospital, Vienna, Austria; 10 Department of Internal Medicine-Haematology and Oncology, University Hospital Brno, Brno, Czech Republic; 11 Department of Internal Medicine-Haematology and Oncology, Masaryk University, Brno, Czech Republic; 12 Department of Paediatrics, Medical University of Vienna, Vienna, Austria

**Keywords:** Invasive fungal disease, panfungal-PCR, Fungal diagnostic, Antifungal therapy, Antifungal treatment

## Abstract

Invasive fungal disease (IFD) presents a life-threatening condition in immunocompromised patients, thus often prompting empirical administration of antifungal treatment, without adequate mycological evidence. Over the past years, wide use of antifungal prophylaxis resulted in decreased occurrence of IFD but has contributed to changes in the spectrum of fungal pathogens, revealing the occurrence of previously rare fungal genera causing breakthrough infections. The expanding spectrum of clinically relevant fungal pathogens required the implementation of screening approaches permitting broad rather than targeted fungus detection to support timely onset of pre-emptive antifungal treatment. To address this diagnostically important aspect in a prospective setting, we analyzed 935 serial peripheral blood (PB) samples from 195 pediatric and adult patients at high risk for IFD, involving individuals displaying febrile neutropenia during treatment of hematological malignancies or following allogeneic hematopoietic stem cell transplantation. Two different panfungal-PCR-screening methods combined with ensuing fungal genus identification by Sanger sequencing were employed. In the great majority of PB-specimens displaying fungal DNAemia, the findings were transient and revealed fungi commonly regarded as non-pathogenic or rarely pathogenic even in the highly immunocompromised patient setting. Hence, to adequately exploit the diagnostic potential of panfungal-PCR approaches for detecting IFD, particularly if caused by hitherto rarely observed fungal pathogens, it is necessary to confirm the findings by repeated testing and to identify the fungal genus present by ensuing analysis. If applied appropriately, panfungal-PCR-screening can help prevent unnecessary empirical therapy, and conversely, contribute to timely employment of effective pre-emptive antifungal treatment strategies.

**Supplementary Information:**

The online version contains supplementary material available at 10.1186/s13045-024-01583-0.

To the Editor:

To account for the diagnostic need to rapidly identify any clinically relevant fungal pathogens, including also previously rare genera causing breakthrough infections [1], we employed two broad-spectrum screening methods previously established in our laboratory, referred to as panfungal-PCR and ITS2-PCR (see Supplementary Material), in combination with ensuing identification of the detected fungal genera by Sanger sequencing and NCBI BLAST analysis [2, 3]. The assessment of their pathogenicity in the immunocompromised setting (Table 1) was based on a classification approach outlined in the Supplementary Material.

We studied serial PB samples from both pediatric and adult patients at high risk for IFD displaying febrile neutropenia (FN) during the treatment of hematological malignancies or after allogeneic hematopoietic stem cell transplantation (HSCT). The patient characteristics, their underlying diseases, the sample collection schedule, and sample processing are outlined in the Supplementary Material. In total, 935 PB-specimens derived from 315 FN-episodes were amenable for analysis: 145 (15.5%) of the PB-specimens investigated (107 from pediatric and 38 from adult patients) derived from 111 FN-episodes (81 in pediatric and 30 in adult patients) revealed transiently positive findings by the PCR-screening methods employed. Exogenous contamination as a potential cause of PCR-positivity was minimized by the precautions employed [4] (see Supplementary Material).

The most frequently detected fungi included *Malassezia* and *Cladosporium* in 19 (13.1%) and 25 (17.2%) PCR-positive specimens, respectively. While *Malassezia* was more prevalent in the pediatric setting (*n* = 16;15.0% vs. *n* = 3;7.9% in adults; Χ^2^(1) = 1.2;*p* = 0.3;RR = 0.5;95% CI 0.2–1.5), *Cladosporium*-positive specimens were somewhat more common in adult patients (*n* = 9;23.7% vs. *n* = 16;15.0% in children; Χ^2^(1) = 1.5;*p* = 0.2;RR = 1.5;95% CI 0.8–3.2) (Fig. 1). Transient DNAemia indicating the presence of proven or probably pathogenic fungi (Fig. 1; Table 1) was detected in a relatively small number of samples obtained from patients, most of whom were receiving antifungal treatment for clinically suspected IFD. The incidence of proven or probable IFD (see Supplementary Material for the definitions used) was very low in the patient cohorts investigated, which might be attributable to the use of effective antifungal prophylaxis. The molecular fungus screening employed was regarded as experimental and the results were therefore not disclosed to the treating physicians. Most fungal DNA sequences detected in PB specimens in the present study were derived from fungal genera typically associated as symbionts or pathogens with plants, which presumably lack pathogenicity in humans (Fig. 1; Table 1), and several of these patients were receiving empirical antifungal treatment during the FN episodes studied (see Supplementary Material).

Classification of fungi based on their potential pathogenicity in the severely immunocompromised patient setting needs to be interpreted with great caution, because fungi regarded as non-pathogenic or rarely pathogenic have occasionally been associated with invasive infections [5, 6]. It is important to point out, however, that the molecular screening performed only identified transient presence of fungal DNAemia in individual specimens during the FN episodes screened, and the findings generally could not be confirmed in subsequent analyses. Hence, the detection of fungal DNAemia indicating the presence of (potentially) pathogenic fungi in the context of FN episodes in immunocompromised patients must be interpreted with prudence, and confirmation by repeated analyses is indicated.

In summary, we were able to detect transient fungal DNAemia in a proportion of immunocompromised patients including individuals with hematologic malignancies and allogeneic HSCT-recipients. Most of the fungi detected by broad-spectrum fungal screening methods would not be regarded as clinically relevant, although their pathogenic potential in the severely immunocompromised setting cannot be completely excluded. Serial testing for fungal DNAemia in immunocompromised patients at risk for IFD using panfungal-PCR approaches offers the potential for permitting early detection of clinically relevant invasive infections. The broad-spectrum screening assays cover not only well-known pathogenic fungi but detect also hitherto rarely observed, potentially emerging fungal pathogens. By contrast, due to the ability of such assays to capture also transient DNAemia caused by plant-associated fungi presumably lacking pathogenicity in humans, as observed in the present study, the findings need to be confirmed by consecutive analyses and the screening must be coupled with rapid identification of the fungal genus or species present. This should be regarded as a prerequisite for determining the potential clinical relevance of positive test results obtained by panfungal PCR screening approaches. If applied appropriately, diagnostic employment of such molecular screening assays could help prevent unnecessary empirical therapy, and conversely, contribute to timely employment of effective pre-emptive antifungal treatment strategies.

**Table 1:** Fungi detected in the present study and classification of their putative pathogenicity in immunocompromised patients. The fungal genera identified in PB samples from high-risk patients using panfungal screening and sequencing of ITS2-PCR amplicons are listed by decreasing hit numbers in two different databases screened for the association of individual fungi with immunocompromised patients (PubMed) and for their implication in fungal infections in humans (FungiQuest). Interestingly, searches in both databases revealed greatly overlapping results, despite the somewhat different search terms employed. The number of hits resulting from the search parameters outlined in the supplementary methods section reflects the proven, probable, possible or absent pathogenicity in humans according to the assessment parameters employed

Pathogenicity	Fungal genus	Positive samples	FungiQuest Hits	PubMed Hits	Total Hits
Proven	*Aspergillus*	1	114	3238	3352
	*Candida*	1	-	2354	2354
	*Diutina*	5	-	see Candida	see Candida
	*Cryptococcus*	1	-	1380	1380
	*Fusarium*	2	135	468	603
	*Trichosporon*	2	138	281	419
Probable	*Penicillium*	7	9	185	194
	*Saccharomyces*	2	14	162	176
	*Alternaria*	8	32	138	170
	*Exophiala*	3	26	96	122
	*Talaromyces*	1	2	73	75
	*Malassezia*	19	2	60	62
	*Cladosporium*	25	15	43	58
Possible	*Trichoderma*	2	11	32	43
	*Aureobasidium*	4	4	17	21
	*Coprinopsis*	1	6	4	10
	*Debaryomyces*	6	0	8	8
	*Lecitophora*	1	0	5	5
	*Yarrowia*	1	0	5	5
	*Pyrenochaeta*	1	0	4	4
	*Sporobolomyces*	1	0	4	4
	*Botrytis*	1	0	2	2
	*Pithomyces*	1	0	2	2
	*Coniothyrium*	1	0	1	1
	*Diaporthe*	1	0	1	1
	*Epicoccum*	4	0	1	1
	*Peniophora*	3	1	0	1
	*Polyporaceae*	1	0	1	1
Absent	*Armillaria*	1	0	0	0
	*Blumeria*	1	0	0	0
	*Bulleromyces*	1	0	0	0
	*Capnodiales*	1	0	0	0
	*Capronia*	1	0	0	0
	*Ciboria*	1	0	0	0
	*Coniosporium*	1	0	0	0
	*Coprinellus*	1	0	0	0
	*Cylindrobasidium*	1	0	0	0
	*Daedaleopsis*	1	0	0	0
	*Filobasidium*	2	0	0	0
	*Flammulina*	1	0	0	0
	*Ganoderma*	1	0	0	0
	*Hyphodontia*	3	0	0	0
	*Inocybe*	1	0	0	0
	*Itersonilia*	1	0	0	0
	*Knufia*	2	0	0	0
	*Laetiporus*	1	0	0	0
	*Leptosphaeria*	2	0	0	0
	*Microcyclospora*	1	0	0	0
	*Mycena*	1	0	0	0
	*Mycosphaerella*	1	0	0	0
	*Myxotrichum*	2	0	0	0
	*Naevala*	1	0	0	0
	*Peniophorella*	1	0	0	0
	*Plectosphaerella*	1	0	0	0
	*Pyrenophora*	1	0	0	0
	*Rhodocollybia*	1	0	0	0
	*Stemphylium*	1	0	0	0
	*Tetracladium*	1	0	0	0
	*Torula*	1	0	0	0
	*Vuilleminia*	1	0	0	0
	*Wallemia*	1	0	0	0

**Fig. 1:**
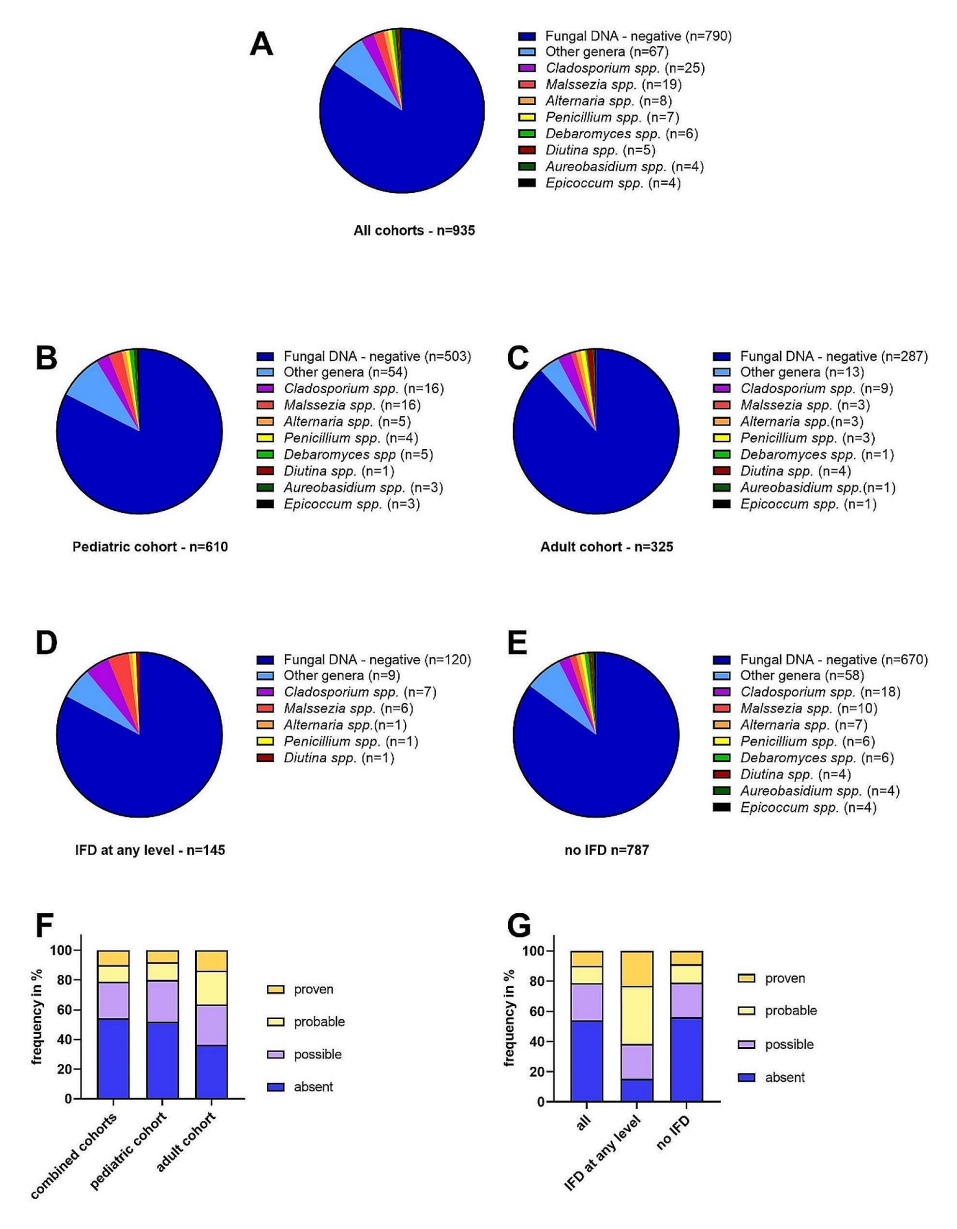
Most commonly detected fungal genera in the cohorts studied and the presumptive pathogenicity of the observed fungal spectrum in the immunocompromised setting. **Panel A**: Overall, almost 85% of the PB samples tested negative by both panfungal-PCR approaches employed. The most commonly observed fungal genus was *Cladosporium*, which was detected in 2.7% of all PB samples tested. **Panels B and C**: The fungal genera detected in pediatric and adult patients revealed no statistically significant differences between the two cohorts, although the genus *Malassezia* was slightly more common in the pediatric setting (2.6% of all PB samples tested in the pediatric versus 0.9% in the adult cohort). **Panels D and E**: The distribution of fungal genera detected in patients with IFD at any level and in patients with absent IFD was very similar. However, the diversity of fungal genera was smaller in the subset of patients with IFD at any level, which could be attributable to the smaller sample size. **Panel F**: Overall, over 50% of the fungal genera identified in PB samples from high-risk patients using panfungal screening and sequencing of ITS2-PCR amplicons were classified as putatively non-pathogenic (indicated as absent pathogenicity in the Figure), when applying the search parameters outlined in the Supplementary Material. The frequency of fungi displaying proven, probable, possible, or absent pathogenicity revealed no statistically significant differences between the pediatric and adult cohorts studied (the numbers for fungal genera with proven pathogenicity were 8.0% vs. 13.6%, *p* = 0.3; with probable pathogenicity 12.0% vs. 22.7%, *p* = 0.1; with possible pathogenicity 28.0% vs. 27.3%, *p* = 0.7; with absent pathogenicity 52.0% vs. 36.4% *p* = 0.6). n, number of PB specimens investigated. **Panel G: **Fungi displaying possible, probable, or proven pathogenicity according to the search parameters applied were observed in 24.6%, 11.5%, and 9.8% of PB samples, when the presence or absence of IFD was not considered (all). However, between patients displaying IFD at any level and patients with absent IFD, there was a statistically significant difference in the frequency of fungal genera with probable pathogenicity (38.5% vs. 12.3%, *p* = 0.02) and absent pathogenicity (15.4% vs. 56.1%; *p *= 0.008). Importantly, however, the fungal DNAemia detected was only transient and could not be confirmed in sequential analyses

### Electronic supplementary material

Below is the link to the electronic supplementary material.

Supplementary Material 1

## Data Availability

The dataset supporting the conclusions of this article is available in the Zenodo repository: https://doi.org/10.5281/zenodo.12795797.

## Declarations

### Clinical trial registration

The study was registered at ClinicalTrials.gov (NCT02492594).
