# Supplementary material for: Prevalence of fungal DNAemia mediated by putatively non-pathogenic fungi in immunocompromised patients with febrile neutropenia: a prospective cohort study
Source: J Hematol Oncol. 2024 Aug 7;17:63. doi: 10.1186/s13045-024-01583-0 (PMC11304904; doi:10.1186/s13045-024-01583-0)
Supplement: Supplementary file 1 — Supplementary Material 1 [file 13045_2024_1583_MOESM1_ESM.pdf]

# Supplementary Material

Patients

In total, 215 immunocompromised patients from a pediatric (n=124) and an adult cohort (n=91) at high risk for IFD were recruited, and 1016 serial PB specimens (n=689 and n=327 from pediatric and adult patients, respectively) were prospectively collected during 376 febrile neutropenic (FN) episodes (n=265 in children and n=111 in adults). The FN episodes with at least two sequential PB specimens amenable to evaluation were considered for further analysis, resulting in a total of 935 PB specimens (n=610 in the pediatric and n=325 in the adult setting) derived from 315 FN episodes (n=205 and n=110, in children and adults, respectively) in 195 patients (n=106 children and n=89 adults) available for comprehensive investigation. The median age of pediatric patients, who were hospitalized at the St. Anna Children's Hospital (Vienna, Austria) or the Princess Máxima Center (Utrecht, the Netherlands), was 6 years (range 0-18; 55% male, 45% female). The underlying diseases in the pediatric patient cohort included acute lymphoblastic leukemia (ALL, 74%), acute myeloid leukemia (AML, 8%), neuroblastoma (4%), lymphoma (3%) and, in rare instances, other malignant disorders (11% in total). The median age of adult patients, who were hospitalized at the Vienna General Hospital (Vienna, Austria), the I. P. Pavlov First Saint Petersburg State Medical University (Saint Petersburg, Russian Federation), or the Hanusch Hospital (Vienna, Austria) was 56 years (range 19-81; 66% male, 34% female). The underlying diseases in the adult cohort included AML (60%), ALL (16%), lymphoma (13%), multiple myeloma (9%) and, in rare instances, other malignant disorders (2%). Diagnoses were established according to the criteria and classification of the World Health Organization (WHO).

Antifungal prophylaxis

In line with the low incidence of IFD at any level, most patients had received antifungal prophylaxis, which was administered before and during the majority of FN episodes analyzed, covering 67.3% (n=74) of episodes in the adult cohort and 82.9 % (n=170) in the pediatric cohort. Information on antifungal prophylaxis in the patients studied is provided in Table S1.

IFD classification

Patients were classified according to the European Organization for Research and Treatment of Cancer/Mycoses Study Group (EORTC/MSG) guidelines[1]. The IFD probability was determined as follows: i) possible IFD (host criterion plus clinical criterion or positive mycological finding), ii) probable (host criterion plus clinical criterion and positive mycological finding), and iii) proven (host criterion plus successful fungus recovery by culture). In the absence of any host or clinical criterion, or the lack of any mycological evidence for an infection, patients were classified as IFD negative, in line with the EORTC/MSG guidelines[1]. Results of the molecular screening methods used in the present study, panfungal PCR and internal transcribed spacer 2 (ITS2) PCR[2,3], were not considered in the classification of IFD.

Sample processing

Serum and PB samples were collected during neutropenic episodes at the onset of fever, and subsequently after 24 and 48 hours. The samples were stored at -80°C until analysis. Serum samples were tested for cell wall antigens (galactomannan and 1,3-β-D-glucan) as described[4]. For molecular testing, 2-3 mL of peripheral blood were treated with DNase to remove free DNA, and blood cells were lysed to isolate intact microorganisms. Samples were spiked with PhHV (phocine herpes virus) DNA as internal control to exclude the presence of inhibitors affecting the efficiency of PCR amplification[5]. Enzymatic and mechanical lysis were combined to achieve efficient release of fungal genomic DNA, which was purified on spin-columns included in the MolYsis™ kit and eluted in 100 µl DNAse-free water. DNA samples were immediately subjected to analysis by panfungal PCR and ITS2 PCR or stored at -80°C until analysis.

Panfungal PCR

Panfungal PCR was performed as described[3]. Briefly, the purified microbial DNA was analyzed by a two-reaction real-time PCR protocol covering a large spectrum of fungi (>80 species) including essentially any fungus with potential relevance in the clinical setting, but also many other fungal species not

associated with pathogenicity in humans. An additional PCR reaction detecting previously spiked PhHV (phocine herpes virus) sequences was used as an internal control for the inhibition of amplification[5]. Primers and probes were designed to detect the 28S gene of moulds (reaction I), or yeasts and Zygomycetes (reaction II), respectively[3]. Each real-time PCR reaction of 25 µl included TaqMan Gene Expression Master mix, primers (4 fmol), TaqMan probe (100 fmol) and DNA template (5 µl). Positive controls (100 fg DNA of *A. fumigatus* and *C. albicans*, respectively), and negative controls (non-template control and water control) were present in each run. Real-time PCR was performed using a TaqMan 7500 Instrument with the standard 9600 Emulation protocol and results were analyzed using the thresholds described in our previous studies[3,5].

<u>ITS2 PCR and sequencing</u>

ITS2 PCR was performed as described previously[2]. Briefly, the purified microbial DNA was amplified in a two-round nested PCR reaction using primers designed to bind within conserved regions flanking the ITS2 region. Amplicons of this highly variable region in the fungal genome were used for identification of the fungal genus or species, if possible. The first PCR reaction contained AmpliTaq DNA Polymerase buffer, $MgCl_2$ (2.5 µM), dNTPs (800 pmol), ITS1 and ITS4 primers (4 fmol), UNG (uracil DNA-glycosylase, 0.5 U), AmpliTaq DNA Polymerase (2.5 U) and DNA template (5 µl) in a volume of 25 µl. The second, nested 50 µl PCR reaction, contained AmpliTaq DNA Polymerase buffer, $MgCl_2$ (2.5 µM), dNTPs (800 pmol), ITS86, ITS86_krusei and ITS4 primers (4 fmol), AmpliTaq DNA Polymerase (2.5 U) and 6 µl of the first-round amplification product as template. Positive controls (100 fg DNA of *A. fumigatus* and *C. albicans*, respectively) and negative controls (non-template control and water control) were present in each run.

<u>Sequencing</u>

Nested PCR products yielding visible bands upon electrophoresis in agarose gels were purified using a Gel extraction kit (Qiagen, Hilden, Germany) and analyzed by Sanger sequencing using primers flanking the highly variable ITS2 region (ITS1 and ITS4)[2]. Sequences were analyzed by NCBI BLAST[6] to facilitate

fungal identification at the genus level. The ITS2 region is highly variable between fungal genera but does not commonly permit identification of fungal species due to insufficient variability at this level.

Prevention of external contamination

The measures employed to prevent exogenous contamination as an occasional source of positive PCR assays included blood collection via central venous catheters under sterile conditions, sample processing in laminar flow cabinets, and the use of multiple negative controls in each step of molecular analysis[7]. Moreover, about 85% of the specimens investigated tested negative for any fungal DNA traces, suggesting that contamination was not a relevant issue.

Classification of fungal genus pathogenicity in immunocompromised patients

While the pathogenicity of certain fungal genera in the immunocompromised setting is well established by experimental and clinical evidence, the clinical relevance of several fungal genera in immunocompromised patients is less clear. It is of note that anecdotal reports have also implicated fungi generally regarded as non-pathogenic in clinically relevant invasive infections in the immunocompromised setting. To assess the reported occurrence of individual fungi in immunocompromised patients, a search in PubMed using the search term "*fungal genus* AND immunocomp* AND patient*" was performed. Additionally, to determine the putative pathogenicity of the spectrum of fungi detected in the patient cohorts investigated in the present study, a query interrogating all fungal genera identified was run using "FungiQuest"[8], a search tool from FungiScope™, which compiles data on the occurrence of fungal infections of less commonly observed fungi in the clinical setting. For the purpose of classification in the present study, the hits of both searching engines were summed and subjected to an internally established classification: all genera above 400 hits were regarded as proven, between 50 and 399 hits as probably pathogenic, between 1 and 49 hits as possibly pathogenic, and those without any hits were considered as fungi with unknown but presumably absent pathogenicity.

Statistical methods

Categorical variables were presented in numbers and percentages. Data were analyzed by Chi square test ($X^2$) or Fisher's exact test using relative risk (RR) for the calculation of the effect size and 95% confidence interval (CI). A *P* value of $< 0.05$ was considered significant. All calculations were done with the Graph Prism 9.3 software.

Occurrence of fungal DNAemia

Analysis of the data derived from patients who had a higher clinical index for IFD, and were classified as proven, probable or possible IFD, revealed no statistically relevant differences in the occurrence of fungal DNAemia to the entire cohort of patients studied: fungal DNAemia was observed in 15.5% in the entire cohort (n=145) versus 17.2% in patients with proven, probable or possible IFD. We have compared the occurrence of fungi displaying different levels of pathogenicity in humans (based on the definition provided) in samples from patients with probable or proven IFD (n=4; 17.4%) versus patients with possible IFD or without any evidence for IFD (n=91; 10.0%), and the data revealed no statistically significant difference between patients displaying different levels of IFD (Fisher's exact test: p=0.3, RR=1.7, 95% CI 0.7-3.8). However, due to the rare occurrence of proven and probable IFD in the patient cohorts studied, the analysis of individual IFD levels is of marginal statistical relevance. The underlying diseases in the patients studied were quite diverse and the number of patients per disease entity was not large enough to perform statistically relevant comparisons. By contrast, when comparing the occurrence of fungal DNAemia between samples from female (n=50; 11.7%) and male (n=95; 18.7%) patients, the higher incidence in males was statistically significant ($X^2(1)=8.8$, p=0.003, RR=0.6, 95% CI 0.5 to 0.9). The significantly higher occurrence of fungal DNAemia observed in male in comparison to female patients was an unexpected finding, although there are hints in the literature indicating a higher susceptibility of males to specific fungal infections involving particularly *Cryptococcus*[9] and *Aspergillus*[10]. However, relevant assessment of the observation made would require multivariate analysis within larger studies.

Potential clinical relevance of rarely pathogenic fungi in the immunocompromised setting

To assess the clinical relevance of the detected fungi in the immunocompromised setting we employed two independent sources, namely PubMed and FungiQuest[8], using the search criteria outlined in the Methods section. While PubMed permits the identification of essentially all publications on fungal infections, FungiQuest focuses particularly on individual, less commonly observed fungi. Although the use of the indicated two sources for the incidence of fungi in the human setting may not provide completely exhaustive data, the probability of overlooking the relevance of a fungal genus is rather low. Nevertheless, a broader search strategy might identify some additional cases of rare fungi occurring as human pathogens. The occurrence of *Malassezia*, a yeast commonly found on human skin and capable of causing invasive infections under certain conditions[12-14], was surprisingly common, particularly in the pediatric patient cohort, and exogenous contamination of the blood samples might offer a possible explanation for this observation. Similarly, *Cladosporium*, the most frequently detected fungal genus in the present study, has been considered a contaminant in some studies[15,16]. However, it may also represent an emerging and potentially pathogenic fungus, as 43 hits were found in PubMed when using the searching algorithm outlined above. Cases of IFD associated with *Cladosporium* in immunocompromised patients were reported in several publications [17-19], and the FungiQuest query tool, representing a registry for emerging fungal infections[8], revealed 15 cases of IFD caused by *Cladosporium* spp. Other examples of fungi with questionable association with pathogenicity in immunocompromised individuals include *Penicillium* spp., known as plant pathogens and allergens in humans, which were mostly considered to be contaminants, but have also been reported to cause IFD in immunocompromised patients[20]. The lack of adequate immune surveillance in severely immunocompromised patients extends the spectrum of fungi capable of causing invasive infections in this setting, and it is difficult therefore to completely exclude individual fungi as potential causes of IFD.

Limitations of the study

Contaminated food or drugs produced by exploiting fungi are well-known sources of circulating fungal DNA in PB [21-23], and this might potentially explain some of the findings in the current study. Semi-synthetic β-lactam antibiotics may contain traces of *Penicillium*, which is involved in the production [24-26]. In the present study, *Penicillium* DNA was identified by ITS2-PCR and sequencing in seven cases, and all these patients were treated with semi-synthetic β-lactam antibiotics at the time of sample collection, but the correlation was not statistically significant.

Similar studies often employed other samples, such as formalin-fixed, paraffin-embedded tissues [27], sterile and nonsterile fluids other than blood[28], different panfungal PCR protocols such as the amplification of the 18S rRNA gene[29] or the ITS1 region[28], thus making a comparison with our results difficult. The reported higher incidences of IFD in some studies correlated with higher detection rates of fungal DNAemia, in the range between 30 and 63%[27-29], while the present study identified fungal DNAemia in only 15% of the samples collected, in line with the low incidence of IFD observed and the common use of antifungal prophylaxis.

While *Candida* and *Aspergillus spp.* have been the most commonly detected fungal pathogens in immunocompromised patients with invasive fungal infection, wide use of broad-spectrum antifungal prophylaxis has resulted in an increasing occurrence of hitherto rarely observed fungal genera. These observations underline the importance of panfungal screening approaches permitting the detection of essentially any fungus of potential clinical relevance [27-29]. However, screening assays involving the use of panfungal PCR approaches are still regarded as experimental, although the diagnostic potential of molecular diagnostics in the immunocompromised setting is well recognized.

As stated above, a major limitation of the present study was the rare occurrence of probable or proven IFD in the patient cohorts investigated, which precluded assessing the full diagnostic potential of broad-spectrum PCR assays, such as the pan-fungal and ITS2 PCR approaches used. Nevertheless, the broad-spectrum screening methods used revealed an unexpectedly high proportion of putatively non-

pathogenic fungi in patients displaying fungal DNAemia, which may not be of clinical relevance even in the severely immunocompromised setting. It is of paramount importance therefore to combine broad-spectrum screening methods with ensuing identification of the detected fungi at the genus or species level and to confirm the results by repeated testing. This in an apparent prerequisite for appropriate interpretation of diagnostic data, which may help preventing unnecessary treatment or providing a basis for appropriate antifungal therapy in clinical practice.

| nr. | Center | P/A | Age (years) | Gender | Underlying disease | IFD scoring | HSCT | Fungal genus (NCBI BLAST) | AB prophylaxis | AB prophylaxis | AB treatment | AB treatment | AM prophylaxis | AM prophylaxis | AM treatment | AM treatment |
|---|---|---|---|---|---|---|---|---|---|---|---|---|---|---|---|---|
| 1 | STA | P | 4 | F | ALL | negative | no | *Malassezia* | yes | Paromomycin | yes | Piperacillin& Tazobactam | yes | Amphotericin B | no | - |
| 2 | STA | P | 16 | M | Burkitt lymphoma | IFD possible | no | *Cladosporium* | yes | Paromomycin | yes | Piperacillin& Tazobactam; Amikacin; Meropenem; Vancomycin | yes | Amphotericin B; Voriconazole | yes | Amphotericin B |
| 2 | STA | P | 16 | M | Burkitt lymphoma | IFD possible | no | *Coniosporium* | yes | Sulfamethoxazole &Trimethoprim | yes | Meropenem | yes | Amphotericin B | no | - |
| 6 | STA | P | 5 | M | ALL | IFD possible | yes | *Hyphodontia* | no | - | yes | Vancomycin | yes | Voriconazole | - | - |
| 7 | STA | P | 1 | F | ALL | negative | no | *Trichoderma* | yes | Paromomycin | yes | Amikacin | yes | Amphotericin B | no | - |
| 7 | STA | P | 1 | F | ALL | negative | no | *Pyrenophora* | yes | Paromomycin; Sulfamethoxazole &Trimethoprim | yes | Amikacin; Piperacillin& Tazobactam | yes | Amphotericin B | no | - |
| 9 | STA | P | 13 | F | ALL | negative | no | *Capronia* | no | - | yes | Piperacillin& Tazobactam | yes | Sulfamethoxazole& Trimethoprim | no | - |
| 10 | STA | P | 5 | F | ALL | IFD possible | no | *Malassezia* | yes | Paromomycin | yes | Amikacin; Piperacillin& Tazobactam | yes | Amphotericin B | no | - |
| 10 | STA | P | 5 | F | ALL | IFD possible | no | *Cryptococcus* | yes | Sulfamethoxazole &Trimethoprim | yes | Amikacin; Piperacillin& Tazobactam | yes | Amphotericin B | no | - |
| 10 | STA | P | 6 | F | ALL | negative | yes | *Epicoccum* | yes | Paromomycin; Sulfamethoxazole &Trimethoprim | yes | Amikacin; Piperacillin& Tazobactam | yes | Amphotericin B | yes | Amphotericin B |
| 11 | STA | P | 3 | M | ALL | IFD possible | no | *Malassezia* | yes | Sulfamethoxazole &Trimethoprim | yes | Amikacin | yes | Amphotericin B | no | - |
| 11 | STA | P | 3 | M | ALL | IFD possible | no | *Malassezia* | yes | Paromomycin | yes | Piperacillin& Tazobactam; Amikacin | yes | Voriconazole | no | - |
| 12 | STA | P | 8 | M | ALL | negative | no | *Exophiala* | no | Paromomycin | yes | Piperacillin& Tazobactam | yes | Amphotericin B | no | - |
| 12 | STA | P | 8 | M | ALL | negative | no | *Cladosporium* | yes | Sulfamethoxazole &Trimethoprim | yes | Amikacin | yes | Amphotericin B; Voriconazole | no | - |
| 12 | STA | P | 9 | M | ALL | negative | no | *Diaporthe* | yes | Paromomycin | yes | Piperacillin& Tazobactam | yes | Amphotericin B | no | - |
| 13 | STA | P | 2 | F | ALL | negative | no | *Penicillium* | yes | Sulfamethoxazole &Trimethoprim | yes | Piperacillin& Tazobactam; Amikacin | yes | Amphotericin B | no | - |
| 13 | STA | P | 3 | F | ALL | negative | no | *Malassezia* | yes | Paromomycin | yes | Piperacillin& Tazobactam | yes | Amphotericin B | no | - |

| nr. | Center | P/A | Age (years) | Gender | Underlying disease | IFD scoring | HSCT | Fungal genus (NCBI BLAST) | AB prophylaxis | AB prophylaxis | AB treatment | AB treatment | AM prophylaxis | AM prophylaxis | AM treatment | AM treatment |
|---|---|---|---|---|---|---|---|---|---|---|---|---|---|---|---|---|
| 15 | STA | P | 13 | M | ALL | negative | no | *Wallemia* | yes | Paromomycin; Sulfamethoxazole &Trimethoprim | yes | Piperacillin& Tazobactam | yes | Amphotericin B | yes | Amphotericin B |
| 16 | STA | P | 5 | M | Renal cell carcinoma | negative | yes | *Malassezia* | no | - | yes | Vancomycin | yes | Sulfamethoxazole&Trimeth oprim | no | - |
| 19 | STA | P | 13 | F | Renal cell carcinoma | negative | yes | *Cladosporium* | no | - | yes | Ceftazidime | yes | Voriconazole | no | - |
| 20 | STA | P | 4 | F | ALL | negative | yes | *Malassezia* | no | - | yes | Piperacillin& Tazobactam ; Amikacin | no | - | yes | Amphotericin B |
| 20 | STA | P | 4 | F | ALL | negative | yes | *Malassezia* | no | - | yes | Meropenem; Vancomycin | no | - | yes | Amphotericin B |
| 21 | STA | P | 11 | M | ALL | negative | no | *Botrytis* | no | - | yes | Piperacillin& Tazobactam | no | - | no | - |
| 22 | STA | P | 12 | M | ALL | IFD possible | yes | *Laetiporus* | no | - | yes | Vancomycin | no | - | no | - |
| 22 | STA | P | 12 | M | ALL | IFD possible | yes | *Ciboria* | no | - | yes | Vancomycin | no | - | yes | Amphotericin B |
| 23 | STA | P | 3 | F | ALL | negative | no | *Malassezia* | yes | Sulfamethoxazole &Trimethoprim | no | - | yes | Amphotericin B | no | - |
| 23 | STA | P | 3 | F | ALL | negative | no | *Knufia* | yes | Paromomycin; Sulfamethoxazole &Trimethoprim | no | - | yes | Amphotericin B | no | - |
| 24 | STA | P | 4 | M | ALL | negative | no | *Malassezia* | yes | Paromomycin | yes | Piperacillin& Tazobactam | yes | Amphotericin B | no | - |
| 24 | STA | P | 4 | M | ALL | negative | no | *Trichosporon* | yes | Sulfamethoxazole &Trimethoprim | yes | Amikacin | yes | Amphotericin B | no | - |
| 24 | STA | P | 4 | M | ALL | negative | no | *Daedaleopsis* | yes | Paromomycin; Sulfamethoxazole &Trimethoprim | yes | Piperacillin& Tazobactam Amikacin | yes | Voriconazole | no | - |
| 25 | STA | P | 17 | F | ALL | negative | no | *Microcyclospora* | yes | Paromomycin | yes | Vancomycin | yes | Amphotericin B | no | - |
| 34 | STA | P | 8 | M | AML | negative | yes | *Talaromyces* | yes | Paromomycin; Sulfamethoxazole &Trimethoprim | yes | Piperacillin& Tazobactam; Vancomycin; Amikacin | yes | Amphotericin B; Voriconazole | no | - |
| 36 | STA | P | 4 | F | ALL | negative | yes | *Malassezia* | yes | Paromomycin; Sulfamethoxazole &Trimethoprim | yes | Piperacillin& Tazobactam; Amikacin | yes | Amphotericin B | yes | Amphotericin B |
| 36 | STA | P | 4 | F | ALL | negative | yes | *Plectosphaerella* | yes | Paromomycin; Sulfamethoxazole &Trimethoprim | yes | Vancomycin; Meropenem | yes | Amphotericin B | yes | Caspofungin |

| nr. | Center | P/A | Age (years) | Gender | Underlying disease | IFD scoring | HSCT | Fungal genus (NCBI BLAST) | AB prophylaxis | AB prophylaxis | AB treatment | AB treatment | AM prophylaxis | AM prophylaxis | AM treatment | AM treatment |
|---|---|---|---|---|---|---|---|---|---|---|---|---|---|---|---|---|
| 40 | STA | P | 1 | M | ALL | negative | no | *Cryptococcus* | yes | Paromomycin; Sulfamethoxazole &Trimethoprim | yes | Amikacin; Piperacillin& Tazobactam; Vancomycin | yes | Amphotericin B | yes | Voriconazole |
| 40 | STA | P | 1 | M | ALL | negative | no | *Peniophora* | yes | Paromomycin; Sulfamethoxazole &Trimethoprim | yes | Amikacin; Piperacillin& Tazobactam; Vancomycin | yes | Amphotericin B | yes | Voriconazole |
| 45 | STA | P | 9 | M | ALL | negative | no | *Cladosporium* | yes | Paromomycin; Sulfamethoxazole &Trimethoprim | yes | Amikacin; Piperacillin& Tazobactam | yes | Amphotericin B; Voriconazole | no | - |
| 45 | STA | P | 9 | M | ALL | negative | no | *Rhodocollybia* | yes | Paromomycin; Sulfamethoxazole &Trimethoprim | yes | Amikacin; Piperacillin& Tazobactam; Teicoplanin | yes | Amphotericin B; Voriconazole | no | - |
| 47 | STA | P | 4 | F | ALL | negative | no | *Peniophorella* | yes | Paromomycin; Sulfamethoxazole &Trimethoprim | yes | Piperacillin& Tazobactam | yes | Amphotericin B | yes | Voriconazole |
| 50 | STA | P | 5 | F | ALL | negative | no | *Coniothyrium* | no | - | yes | Piperacillin& Tazobactam; Amikacin | no | - | yes | Voriconazole |
| 53 | STA | P | 7 | M | ALL | negative | yes | *Inocybe* | yes | Paromomycin; Sulfamethoxazole &Trimethoprim | yes | Amikacin; Meropenem | yes | Amphotericin B | yes | Amphotericin B |
| 56 | STA | P | 4 | M | ALL | negative | no | *Hyphodontia* | yes | Paromomycin; Sulfamethoxazole &Trimethoprim | yes | Piperacillin& Tazobactam; Amikacin | yes | Amphotericin B | no | - |
| 56 | STA | P | 4 | M | ALL | negative | no | *Penicillium* | yes | Paromomycin; Sulfamethoxazole &Trimethoprim | yes | Piperacillin& Tazobactam; Amikacin | yes | Amphotericin B | yes | Amphotericin B |
| 60 | STA | P | 2 | M | T-cell lymphoblastic lymphoma | negative | no | *Debaryomyces* | yes | Sulfamethoxazole &Trimethoprim; Paromomycin | yes | Piperacillin& Tazobactam; Amikacin | yes | Amphotericin B | no | - |
| 60 | STA | P | 2 | M | T-cell lymphoblastic lymphoma | negative | no | *Cladosporium* | yes | Sulfamethoxazole &Trimethoprim; Paromomycin | yes | Piperacillin& Tazobactam; Amikacin | yes | Amphotericin B | no | - |
| 60 | STA | P | 2 | M | T-cell lymphoblastic lymphoma | negative | no | *Debaryomyces* | yes | Sulfamethoxazole &Trimethoprim; Paromomycin | yes | Piperacillin& Tazobactam; Amikacin | yes | Amphotericin B | no | - |
| 61 | STA | P | 3 | F | ALL | negative | no | *Alternaria* | yes | Paromomycin; Sulfamethoxazole &Trimethoprim | yes | Piperacillin& Tazobactam | yes | Amphotericin B | no | - |

| nr. | Center | P/A | Age (years) | Gender | Underlying disease | IFD scoring | HSCT | Fungal genus (NCBI BLAST) | AB prophylaxis | AB prophylaxis | AB treatment | AB treatment | AM prophylaxis | AM prophylaxis | AM treatment | AM treatment |
|---|---|---|---|---|---|---|---|---|---|---|---|---|---|---|---|---|
| 63 | STA | P | 4 | M | ALL | negative | no | *Leptosphaeria* | yes | Sulfamethoxazole &Trimethoprim; Paromomycin | yes | Piperacillin& Tazobactam | yes | Amphotericin B | no | - |
| 63 | STA | P | 4 | M | ALL | negative | no | *Malassezia* | yes | Sulfamethoxazole &Trimethoprim; Paromomycin | yes | Piperacillin& Tazobactam; Amikacin | yes | Amphotericin B | no | - |
| 64 | STA | P | 2 | F | ALL | negative | no | *Debaryomyces* | yes | Sulfamethoxazole &Trimethoprim; Paromomycin | yes | Piperacillin& Tazobactam; Amikacin | yes | Amphotericin B | no | - |
| 65 | STA | P | 7 | M | ALL | negative | no | *Malassezia* | yes | Paromomycin; Sulfamethoxazole &Trimethoprim | yes | Amikacin; Piperacillin& Tazobactam | yes | Amphotericin B | no | - |
| 65 | STA | P | 7 | M | ALL | negative | no | *Alternaria* | yes | Paromomycin; Sulfamethoxazole &Trimethoprim | yes | Amikacin; Piperacillin& Tazobactam | yes | Amphotericin B | no | - |
| 66 | STA | P | 2 | M | ALL | negative | no | *Yarrowia* | yes | Paromomycin; Sulfamethoxazole &Trimethoprim | yes | Piperacillin& Tazobactam | yes | Amphotericin B | no | - |
| 67 | STA | P | 14 | F | ALL | negative | yes | *Pithomyces* | yes | Paromomycin; Sulfamethoxazole &Trimethoprim | yes | Piperacillin& Tazobactam; Teicoplanin | yes | Amphotericin B | yes | Voriconazole |
| 69 | STA | P | 15 | F | ALL | negative | no | *Ganoderma* | yes | Sulfamethoxazole &Trimethoprim; Paromomycin | yes | Piperacillin& Tazobactam; Amikacin | yes | Amphotericin B | no | - |
| 70 | STA | P | 4 | M | ALL | negative | no | *Filobasidium* | yes | Paromomycin | yes | Piperacillin& Tazobactam; Amikacin | yes | Amphotericin B | no | - |
| 70 | STA | P | 4 | F | ALL | negative | no | *Malassezia* | yes | Paromomycin | yes | Piperacillin& Tazobactam; Amikacin | yes | Amphotericin B | no | - |
| 72 | STA | P | 9 | F | ALL | negative | no | *Aureobasidium* | yes | Sulfamethoxazole &Trimethoprim | yes | Piperacillin& Tazobactam; Amikacin | no | - | yes | Voriconazole |
| 73 | STA | P | 7 | M | ALL | negative | no | *Penicillium* | yes | Paromomycin; Sulfamethoxazole &Trimethoprim | yes | Piperacillin& Tazobactam; Amikacin | yes | Amphotericin B | no | - |
| 74 | STA | P | 3 | F | ALL | negative | no | *Cryptococcus* | yes | Paromomycin; Sulfamethoxazole &Trimethoprim | yes | Piperacillin& Tazobactam | yes | Amphotericin B | no | - |
| 75 | STA | P | 1 | M | AML | negative | no | *Cladosporium* | yes | Sulfamethoxazole &Trimethoprim | yes | Amikacin; Piperacillin& Tazobactam; Teicoplanin | yes | Amphotericin B | yes | Amphotericin B |

| nr. | Center | P/A | Age (years) | Gender | Underlying disease | IFD scoring | HSCT | Fungal genus (NCBI BLAST) | AB prophylaxis | AB prophylaxis | AB treatment | AB treatment | AM prophylaxis | AM prophylaxis | AM treatment | AM treatment |
|---|---|---|---|---|---|---|---|---|---|---|---|---|---|---|---|---|
| 75 | STA | P | 1 | M | AML | negative | no | *Cladosporium* | yes | Sulfamethoxazole &Trimethoprim | yes | Piperacillin& Tazobactam; Teicoplanin; Amikacin | yes | Amphotericin B | yes | Amphotericin B |
| 75 | STA | P | 1 | M | AML | negative | no | *Blumeria* | yes | Sulfamethoxazole &Trimethoprim | yes | Meropenem; Amikacin; Teicoplanin | yes | Amphotericin B | yes | Amphotericin B |
| 75 | STA | P | 1 | M | AML | negative | no | *Alternaria* | yes | Sulfamethoxazole &Trimethoprim | yes | Piperacillin& Tazobactam | yes | Amphotericin B | yes | Amphotericin B |
| 75 | STA | P | 1 | M | AML | negative | no | *Cladosporium* | yes | Sulfamethoxazole &Trimethoprim | yes | Piperacillin& Tazobactam; Teicoplanin; Amikacin | yes | Amphotericin B | yes | Amphotericin B |
| 76 | STA | P | 3 | M | ALL | negative | yes | *Torula* | yes | Sulfamethoxazole &Trimethoprim | yes | Ceftriaxone | yes | Voriconazole | no | - |
| 77 | STA | P | 11 | M | ALL | negative | no | *Armillaria* | yes | Sulfamethoxazole &Trimethoprim; Paromomycin | no | - | yes | Amphotericin B | no | - |
| 78 | STA | P | 4 | M | ALL | negative | no | *Bulleromyces* | yes | Sulfamethoxazole &Trimethoprim; Paromomycin | yes | Teicoplanin; Piperacillin& Tazobactam; Amikacin | yes | Amphotericin B | yes | Amphotericin B |
| 78 | STA | P | 4 | M | ALL | negative | no | *Filobasidium* | n.a. | - | n.a. | n.a. | n.a. | n.a. | n.a. | n.a. |
| 78 | STA | P | 4 | M | ALL | negative | no | *Aureobasidium* | yes | Sulfamethoxazole &Trimethoprim; Paromomycin | yes | Teicoplanin | yes | Amphotericin B | yes | Amphotericin B |
| 79 | STA | P | 14 | M | Burkitt lymphoma | negative | no | *Cladosporium* | yes | Sulfamethoxazole &Trimethoprim; Paromomycin | no | - | yes | Amphotericin B | no | - |
| 80 | STA | P | 3 | M | ALL | negative | no | *Cladosporium* | no | - | yes | Piperacillin& Tazobactam; Amikacin | no | - | no | - |
| 81 | STA | P | 18 | M | AML | negative | no | *Myxotrichum* | yes | Sulfamethoxazole &Trimethoprim; Paromomycin; Teicoplanin | yes | Meropenem; Amikacin | yes | Amphotericin B | yes | Fluconazole; Voriconazole |
| 82 | STA | P | 6 | M | ALL | negative | no | *Cladosporium* | yes | Sulfamethoxazole &Trimethoprim; Paromomycin | yes | Meropenem; Vancomycin | yes | Amphotericin B | yes | Voriconazole; Amphotericin B |
| 82 | STA | P | 6 | M | ALL | negative | no | *Sporobolomyces* | yes | Sulfamethoxazole &Trimethoprim; Paromomycin | yes | Meropenem; Vancomycin | yes | Amphotericin B | yes | Voriconazole; Amphotericin B |

| nr. | Center | P/A | Age (years) | Gender | Underlying disease | IFD scoring | HSCT | Fungal genus (NCBI BLAST) | AB prophylaxis | AB prophylaxis | AB treatment | AB treatment | AM prophylaxis | AM prophylaxis | AM treatment | AM treatment |
|---|---|---|---|---|---|---|---|---|---|---|---|---|---|---|---|---|
| 82 | STA | P | 6 | M | ALL | negative | no | *Alternaria* | yes | Sulfamethoxazole &Trimethoprim; Paromomycin | yes | Piperacillin& Tazobactam; Amikacin | yes | Amphotericin B | no | - |
| 82 | STA | P | 6 | M | ALL | negative | no | *Cryptococcus* | yes | Sulfamethoxazole &Trimethoprim; Paromomycin | yes | Amikacin | yes | Amphotericin B | no | - |
| 83 | STA | P | 6 | M | ALL | negative | no | *Cryptococcus* | yes | Sulfamethoxazole &Trimethoprim; Paromomycin | yes | Amikacin | yes | Amphotericin B | no | - |
| 83 | STA | P | 6 | M | ALL | negative | no | *Epicoccum* | yes | Sulfamethoxazole &Trimethoprim; Paromomycin | yes | Piperacillin& Tazobactam; Amikacin | yes | Amphotericin B | yes | Voriconazole |
| 83 | STA | P | 7 | M | ALL | negative | no | *Myxotrichum* | yes | Sulfamethoxazole &Trimethoprim; Paromomycin | yes | Clarithromycin; Meropenem; Teicoplanin | yes | Amphotericin B | yes | Voriconazole |
| 83 | STA | P | 6 | M | ALL | negative | no | *Leptosphaeria* | yes | Sulfamethoxazole &Trimethoprim; Paromomycin | yes | Teicoplanin; Piperacillin& Tazobactam; Amikacin | yes | Amphotericin B | yes | Voriconazole |
| 84 | STA | P | 9 | F | ALL | negative | no | *Itersonilia* | yes | Sulfamethoxazole &Trimethoprim; Paromomycin | yes | Piperacillin& Tazobactam; Amikacin | yes | Amphotericin B | yes | Amphotericin B |
| 84 | STA | P | 9 | F | ALL | negative | no | *Coprinopsis* | yes | Sulfamethoxazole &Trimethoprim; Paromomycin | yes | Piperacillin& Tazobactam; Amikacin | yes | Amphotericin B | yes | Amphotericin B |
| 84 | STA | P | 9 | F | ALL | negative | no | *Cladosporium* | yes | Sulfamethoxazole &Trimethoprim; Paromomycin | yes | Piperacillin& Tazobactam; Amikacin | yes | Amphotericin B | yes | Amphotericin B |
| 84 | STA | P | 9 | F | ALL | negative | no | *Debaryomyces* | yes | Sulfamethoxazole &Trimethoprim; Paromomycin | yes | Piperacillin& Tazobactam | yes | Amphotericin B | no | - |
| 84 | STA | P | 9 | F | ALL | negative | no | *Aureobasidium* | yes | Sulfamethoxazole &Trimethoprim; Paromomycin | yes | Piperacillin& Tazobactam | yes | Amphotericin B | no | - |
| 86 | STA | P | 12 | M | ALL | negative | yes | *Cladosporium* | yes | Sulfamethoxazole &Trimethoprim; Paromomycin | yes | Amikacin; Teicoplanin | yes | Amphotericin B | no | - |
| 87 | STA | P | 4 | M | ALL | negative | yes | *Alternaria* | yes | Sulfamethoxazole &Trimethoprim; Paromomycin | no | - | yes | Amphotericin B | yes | Voriconazole |
| 88 | STA | P | 3 | M | ALL | negative | no | *Epicoccum* | yes | Sulfamethoxazole &Trimethoprim; Paromomycin | no | - | yes | Amphotericin B | no | - |

| nr. | Center | P/A | Age (years) | Gender | Underlying disease | IFD scoring | HSCT | Fungal genus (NCBI BLAST) | AB prophylaxis | AB prophylaxis | AB treatment | AB treatment | AM prophylaxis | AM prophylaxis | AM treatment | AM treatment |
|---|---|---|---|---|---|---|---|---|---|---|---|---|---|---|---|---|
| 89 | STA | P | 12 | M | AML | negative | no | *Cladosporium* | yes | Sulfamethoxazole &Trimethoprim; Paromomycin | yes | Teicoplanin; Piperacillin& Tazobactam; Amikacin | yes | Amphotericin B | yes | Voriconazole |
| 90 | STA | P | 4 | F | ALL | negative | no | *Flammulina* | yes | Sulfamethoxazole &Trimethoprim; Paromomycin | yes | Piperacillin& Tazobactam | yes | Amphotericin B | no | - |
| 91 | STA | P | 2 | M | ALL | negative | no | *Peniophora* | yes | Sulfamethoxazole &Trimethoprim; Paromomycin | yes | Piperacillin& Tazobactam | yes | Amphotericin B | no | - |
| 91 | STA | P | 2 | M | ALL | negative | no | *Exophiala* | yes | Sulfamethoxazole &Trimethoprim; Paromomycin | yes | Piperacillin& Tazobactam | yes | Amphotericin B | no | - |
| 92 | STA | P | 2 | F | ALL | negative | no | *Debaryomyces* | yes | Sulfamethoxazole &Trimethoprim; Paromomycin | yes | Piperacillin& Tazobactam; Amikacin | yes | Amphotericin B | no | - |
| 93 | STA | P | 17 | M | ALL | negative | no | *Exophiala* | yes | Sulfamethoxazole &Trimethoprim; Paromomycin | yes | Piperacillin& Tazobactam; Amikacin | yes | Amphotericin B | no | - |
| 93 | STA | P | 17 | M | ALL | negative | no | *Tetracladium* | yes | Sulfamethoxazole &Trimethoprim; Paromomycin | yes | Piperacillin& Tazobactam; Amikacin | yes | Amphotericin B | no | - |
| 95 | STA | P | 3 | M | ALL | negative | no | *Knufia* | yes | Paromomycin; Pentacarinat | yes | Piperacillin& Tazobactam; Amikacin | yes | Amphotericin B | yes | Amphotericin B |
| 95 | STA | P | 3 | M | ALL | negative | no | *Coprinellus* | yes | Paromomycin; Pentacarinat | yes | Piperacillin& Tazobactam; Amikacin | yes | Amphotericin B | yes | Amphotericin B |
| 1 | UTR | P | 4 | M | Neuroblastoma | negative | YES | *Trichoderma* | yes | Ciprofloxacin | yes | Ceftazidime | yes | Itraconazole | yes | Miconazole |
| 1 | UTR | P | 4 | M | Neuroblastoma | negative | YES | *Malassezia* | yes | Ciprofloxacin | yes | Colistin | yes | Itraconazole | yes | Miconazole |
| 2 | UTR | P | 10 | M | Rhabdoid stomach tumor | negative | no | *Cladosporium* | - | - | yes | Amoxicillin; Gentamicin | yes | Voriconazole | no | - |
| 3 | UTR | P | 9 | M | Burkitt lymphoma | negative | no | *Penicillium* | yes | Ciprofloxacin | yes | Amoxicillin; Gentamicin | yes | Itraconazole | no | - |
| 3 | UTR | P | 9 | M | Burkitt lymphoma | negative | no | *Candida* | yes | Ciprofloxacin | yes | Amoxicillin; Gentamicin | yes | Itraconazole | no | - |
| 5 | UTR | P | 16 | F | ALL | IFD probable | no | *Malassezia* | yes | Sulfamethoxazole &Trimethoprim | yes | Ceftazidime | yes | Itraconazole | no | - |
| 5 | UTR | P | 16 | F | ALL | IFD probable | no | *Diutina* | yes | Sulfamethoxazole &Trimethoprim | yes | Azithromycin | yes | Itraconazole | no | - |
| 7 | UTR | P | 11 | M | ALL | negative | no | *Cladosporium* | yes | Ciprofloxacin; Sulfamethoxazole &Trimethoprim | yes | Ceftazidime | yes | Itraconazole | no | - |

| nr. | Center | P/A | Age (years) | Gender | Underlying disease | IFD scoring | HSCT | Fungal genus (NCBI BLAST) | AB prophylaxis | AB prophylaxis | AB treatment | AB treatment | AM prophylaxis | AM prophylaxis | AM treatment | AM treatment |
|---|---|---|---|---|---|---|---|---|---|---|---|---|---|---|---|---|
| 8 | UTR | P | 2 | F | Neuroblastoma | negative | no | *Mycena* | yes | Ciprofloxacin | yes | Meropenem | yes | Itraconazole | no | - |
| 11 | AKH | A | 48 | M | Multiple myeloma | IFD possible | yes | *Alternaria* | no | - | yes | Cefepime | yes | Fluconazole | no | - |
| 12 | AKH | A | 54 | M | ALL | IFD possible | no | *Malassezia* | yes | Trimethoprim | yes | Piperacillin& Tazobactam | no | - | no | - |
| 12 | AKH | A | 54 | M | ALL | IFD possible | no | *Fusarium* | yes | Trimethoprim | yes | Piperacillin& Tazobactam | no | - | no | - |
| 24 | AKH | A | 46 | M | ALL | IFD possible | no | *Polyporaceae* | no | - | yes | Piperacillin& Tazobactam | yes | Fluconazole | no | - |
| 25 | AKH | A | 79 | F | ALL | IFD possible | no | *Malassezia* | yes | Ciprofloxacin | yes | Piperacillin& Tazobactam | yes | Fluconazole | no | - |
| 25 | AKH | A | 79 | F | ALL | IFD possible | no | *Mycosphaerella* | no | - | yes | Piperacillin& Tazobactam | yes | Fluconazole | no | - |
| 26 | AKH | A | 74 | F | AML | negative | no | *Alternaria* | no | - | yes | Piperacillin& Tazobactam Moxifloxacin | yes | Fluconazole | no | - |
| 27 | AKH | A | 44 | F | T-cell lymphoma | negative | no | *Penicillium* | yes | Ciprofloxacin | yes | Piperacillin& Tazobactam | yes | Fluconazole | yes | Amphotericin B; Fluconazole |
| 28 | AKH | A | 66 | M | AML | negative | no | *Penicillium* | yes | - | yes | Piperacillin& Tazobactam | yes | Posaconazole | no | - |
| 29 | AKH | A | 54 | M | ALL | negative | no | *Capnoidales* | no | - | yes | Piperacillin& Tazobactam | no | - | no | - |
| 30 | AKH | A | 58 | F | ALL | IFD possible | no | *Penicillium* | yes | Ciprofloxacin | yes | Cefepime | yes | Fluconazole | no | - |
| 34 | AKH | A | 63 | F | AML | IFD possible | no | *Cladosporium* | yes | - | no | - | yes | Fluconazole | no | - |
| 40 | AKH | A | 38 | M | AML | negative | no | *Lecythophora* | no | - | yes | Piperacillin& Tazobactam | yes | Posaconazole | no | - |
| 40 | AKH | A | 38 | M | AML | negative | no | *Alternaria* | no | - | yes | Piperacillin& Tazobactam | no | - | no | - |
| 41 | AKH | A | 25 | F | AML | negative | no | *Trichosporon* | yes | Trimethoprim | no | - | no | - | no | - |
| 41 | AKH | A | 25 | F | AML | negative | no | *Epicoccum* | no | - | yes | Piperacillin& Tazobactam | no | - | no | - |
| 41 | AKH | A | 25 | F | AML | negative | no | *Aureobasidium* | yes | Ciprofloxacin | yes | Piperacillin& Tazobactam | yes | Itraconazole | no | - |
| 47 | AKH | A | 26 | M | AML | negative | no | *Cladosporium* | yes | Levofloxacin | yes | Piperacillin& Tazobactam | yes | Posaconazole | no | - |
| 47 | AKH | A | 26 | M | AML | negative | no | *Stemphylium* | yes | Levofloxacin | yes | Piperacillin& Tazobactam | yes | Posaconazole | no | - |
| 48 | AKH | A | 30 | M | ALL | IFD possible | no | *Saccharomyces* | no | - | yes | Piperacillin& Tazobactam | no | - | no | - |
| 54 | AKH | A | 24 | F | Burkitt lymphoma | negative | no | *Debaromyces* | no | - | yes | Piperacillin& Tazobactam | no | - | no | - |

| nr. | Center | P/A | Age (years) | Gender | Underlying disease | IFD scoring | HSCT | Fungal genus (NCBI BLAST) | AB prophylaxis | AB prophylaxis | AB treatment | AB treatment | AM prophylaxis | AM prophylaxis | AM treatment | AM treatment |
|---|---|---|---|---|---|---|---|---|---|---|---|---|---|---|---|---|
| 55 | AKH | A | 61 | M | Multiple myeloma | negative | yes | *Fusarium* | no | - | yes | Piperacillin& Tazobactam | no | - | no | - |
| 1 | HAN | A | 73 | M | Multiple myeloma | IFD possible | yes | *Cladosporium* | no | - | yes | Linezolid; Meropenem | yes | Amphotericin B | yes | Voriconazole; Amphotericin B |
| 1 | HAN | A | 73 | M | Multiple myeloma | IFD possible | yes | *Hypodonthia* | no | - | yes | Linezolid; Meropenem | yes | Amphotericin B | yes | Voriconazole; Amphotericin B |
| 2 | HAN | A | 71 | M | AML | IFD possible | no | *Cladosporium* | no | - | yes | Piperacillin& Tazobactam | yes | Amphotericin B; Posaconazole | no | - |
| 3 | HAN | A | 68 | F | AML | negative | no | *Cladosporium* | yes | Trimethoprim | yes | Teicoplanin | yes | Posaconazole | no | - |
| 7 | HAN | A | 58 | M | AML | negative | no | *Aspergillus* | no | - | yes | Amikacin; Piperacillin& Tazobactam | yes | Amphotericin B inhalation; Amphotericin B | no | - |
| 9 | HAN | A | 58 | M | CML | negative | no | *Candida* | no | - | yes | Vancomycin; Meropenem | yes | Posaconazole | no | - |
| 13 | HAN | A | 36 | M | AML | negative | no | *Peniophora* | yes | Trimethoprim | yes | Meropenem | yes | Amphotericin B inhalation | no | - |
| 17 | HAN | A | 64 | M | AML | negative | no | *Saccharomyces* | yes | Trimethoprim | yes | Piperacillin& Tazobactam | yes | Amphotericin B inhalation | no | - |
| 20 | HAN | A | 66 | M | Multiple myeloma | IFD probable | yes | *Cladosporium* | yes | Trimethoprim | yes | Teicoplanin; Cefepime | yes | Amphotericin B inhalation | yes | Amphotericin B |
| 1 | STP | A | 59 | M | AML | IFD proven | no | *Cladosporium* | yes | Sulfamethoxazole &Trimethoprim | yes | Meropenem | no | - | no | - |
| 2 | STP | A | 32 | M | AML | IFD possible | yes | *Vuilleminia* | yes | Sulfamethoxazole &Trimethoprim | yes | Cefoperazone& Sulbactam | no | - | no | - |
| 2 | STP | A | 32 | M | AML | IFD possible | yes | *Cladosporium* | yes | Sulfamethoxazole &Trimethoprim | yes | Cefoperazone& Sulbactam | no | - | no | - |
| 3 | STP | A | 27 | F | AML | negative | yes | *Naevala* | yes | Sulfamethoxazole &Trimethoprim | yes | Meropenem; Tigecycline | no | - | no | - |
| 5 | STP | A | 22 | M | T-cell lymphoma | negative | yes | *Malassezia* | yes | Sulfamethoxazole &Trimethoprim | yes | Meropenem | no | - | no | - |
| 5 | STP | A | 22 | M | T-cell lymphoma | negative | yes | *Cladosporium* | yes | Sulfamethoxazole &Trimethoprim | yes | Meropenem | no | - | no | - |
| 6 | STP | A | 43 | F | ALL | negative | yes | *Cylindrobasidium* | yes | Sulfamethoxazole &Trimethoprim | yes | Meropenem | no | - | no | - |
| 13 | STP | A | 26 | M | AML | negative | yes | *Pyrenochaeta* | yes | Sulfamethoxazole &Trimethoprim | yes | Meropenem; Vancomycin | no | - | no | - |

**Table S1. Patient characteristics**

Abbreviations: nr., patient number; P/A, pediatric/adult; F, female; M, male; STA, St. Anna Children´s Hospital, Vienna, Austria; UTR, Princess Máxima Center, Utrecht, The Netherlands; AKH, Vienna General Hospital, Vienna, Austria); HAN, Hanusch Hospital, Vienna, Austria; STP, I. P. Pavlov First Saint Petersburg State Medical University, Saint Petersburg, Russian Federation; AB, antibiotic; AM, antimycotic.

## REFERENCES


1. Donnelly JP, Chen SC, Kauffman CA, et al. Revision and Update of the Consensus Definitions of Invasive Fungal Disease From the European Organization for Research and Treatment of Cancer and the Mycoses Study Group Education and Research Consortium. *Clin Infect Dis.* 2020;71(6):1367-1376.
2. Landlinger C, Baskova L, Preuner S, Willinger B, Buchta V, Lion T. Identification of fungal species by fragment length analysis of the internally transcribed spacer 2 region. *Eur J Clin Microbiol Infect Dis.* 2009;28(6):613-622.
3. Landlinger C, Preuner S, Baskova L, et al. Diagnosis of invasive fungal infections by a real-time panfungal PCR assay in immunocompromised pediatric patients. *Leukemia.* 2010;24(12):2032-2038.
4. Weinbergerova B, Kocmanova I, Racil Z, Mayer J. Serological Approaches. In: Lion T, ed. *Human Fungal Pathogen Identification: Methods and Protocols.* New York, NY: Springer New York; 2017:209-221.
5. Czurda S, Lion T. Broad-Spectrum Molecular Detection of Fungal Nucleic Acids by PCR-Based Amplification Techniques. *Methods Mol Biol.* 2017;1508:257-266.
6. Altschul SF, Gish W, Miller W, Myers EW, Lipman DJ. Basic local alignment search tool. *J Mol Biol.* 1990;215(3):403-410.
7. Czurda S, Lion T. Prerequisites for Control of Contamination in Fungal Diagnosis. *Methods Mol Biol.* 2017;1508:249-255.
8. Seidel D, Duran Graeff LA, Vehreschild M, et al. FungiScope() -Global Emerging Fungal Infection Registry. *Mycoses.* 2017;60(8):508-516.
9. Guess TE, Rosen JA, McClelland EE. An Overview of Sex Bias in C. neoformans Infections. *J Fungi (Basel).* 2018;4(2).
10. Sun KS, Tsai CF, Chen SC, Chen YY, Huang WC. Galactomannan Testing and the Incidence of Invasive Pulmonary Aspergillosis: A 10-Year Nationwide Population-Based Study in Taiwan. *PLoS One.* 2016;11(2):e0149964.
11. Chiara Cattaneo MB, Nicola Fracchiolla, Federica Gigli, Claudia Basilico, Lorenzo Masina, Erika Borlenghi, Alessandro Bruno, Giselda Gela, Diego Bertoli, Giuseppe Rossi, Alessandra Tucci, Federico Lussana, Elisabetta Todisco. Different Epidemiology and Characteristics of Invasive Pulmonary Aspergillosis in Acute Lymphoid Leukemia in Comparison with AML: Results of a Prospective Multicentric Observational Study of the Rete Ematologica Lombarda (REL). *Blood.* 2023;142.
12. Golan Y. Overview of transplant mycology. *Am J Health Syst Pharm.* 2005;62(8 Suppl 1):S17-21.
13. Pfaller MA, Diekema DJ, Merz WG. Chapter 10 - Infections caused by non-Candida, non-Cryptococcus yeasts. In: Anaissie EJ, McGinnis MR, Pfaller MA, eds. *Clinical Mycology (Second Edition).* Edinburgh: Churchill Livingstone; 2009:251-270.
14. Rhimi W, Theelen B, Boekhout T, Otranto D, Cafarchia C. Malassezia spp. Yeasts of Emerging Concern in Fungemia. *Front Cell Infect Microbiol.* 2020;10:370.
15. Camp I, Manhart G, Schabereiter-Gurtner C, Spettel K, Selitsch B, Willinger B. Clinical evaluation of an in-house panfungal real-time PCR assay for the detection of fungal pathogens. *Infection.* 2020;48(3):345-355.
16. Comacle P, Belaz S, Jegoux F, et al. Contribution of molecular tools for the diagnosis and epidemiology of fungal chronic rhinosinusitis. *Med Mycol.* 2016;54(8):794-800.
17. Sproson EL, Thomas KM, Lau LC, Harries PG, Howarth PH, Salib RJ. Common airborne fungi induce species-specific effects on upper airway inflammatory and remodelling responses. *Rhinology.* 2016;54(1):51-55.
18. Villanueva DM, Venkatesan B, Figueroa N. Cladosporium sphaerospermum as a Rare Cause of Pneumonia. *Cureus.* 2022;14(6):e26256.

19. Yano S, Koyabashi K, Kato K. Intrabronchial lesion due to Cladosporium sphaerospermum in a healthy, non-asthmatic woman. *Mycoses.* 2003;46(8):348-350.
20. Hoenigl M, Salmanton-Garcia J, Walsh TJ, et al. Global guideline for the diagnosis and management of rare mould infections: an initiative of the European Confederation of Medical Mycology in cooperation with the International Society for Human and Animal Mycology and the American Society for Microbiology. *Lancet Infect Dis.* 2021;21(8):e246-e257.
21. Ghannoum M. Azole Resistance in Dermatophytes: Prevalence and Mechanism of Action. *J Am Podiatr Med Assoc.* 2016;106(1):79-86.
22. Guigue N, Alanio A, Menotti J, et al. Utility of adding Pneumocystis jirovecii DNA detection in nasopharyngeal aspirates in immunocompromised adult patients with febrile pneumonia. *Med Mycol.* 2015;53(3):241-247.
23. Stosor V, Zembower, T. R. . Infectious Complications in Cancer Patients. *Anticancer Research.* 2015;35(1):593-593.
24. Pinel C, Fricker-Hidalgo H, Lebeau B, et al. Detection of circulating Aspergillus fumigatus galactomannan: value and limits of the Platelia test for diagnosing invasive aspergillosis. *J Clin Microbiol.* 2003;41(5):2184-2186.
25. Sulahian A, Touratier S, Ribaud P. False positive test for aspergillus antigenemia related to concomitant administration of piperacillin and tazobactam. *N Engl J Med.* 2003;349(24):2366-2367.
26. Walsh TJ, Groll A, Hiemenz J, Fleming R, Roilides E, Anaissie E. Infections due to emerging and uncommon medically important fungal pathogens. *Clin Microbiol Infect.* 2004;10 Suppl 1:48-66.
27. Gomez CA, Budvytiene I, Zemek AJ, Banaei N. Performance of Targeted Fungal Sequencing for Culture-Independent Diagnosis of Invasive Fungal Disease. *Clin Infect Dis.* 2017;65(12):2035-2041.
28. Trubiano JA, Dennison AM, Morrissey CO, et al. Clinical utility of panfungal polymerase chain reaction for the diagnosis of invasive fungal disease: a single center experience. *Med Mycol.* 2016;54(2):138-146.
29. Sugawara Y, Nakase K, Nakamura A, et al. Clinical utility of a panfungal polymerase chain reaction assay for invasive fungal diseases in patients with haematologic disorders. *Eur J Haematol.* 2013;90(4):331-339.
